# The speciation of inorganic arsenic in soil and vegetables irrigated with treated municipal wastewater

**DOI:** 10.1039/c9ra08031g

**Published:** 2020-01-08

**Authors:** Mari Ataee, Toraj Ahmadi-Jouibari, Negar Noori, Nazir Fattahi

**Affiliations:** Clinical Research Development Center, Imam Khomeini and Mohammad Kermanshahi and Farabi Hospitals, Kermanshah University of Medical Sciences Kermanshah Iran; Research Center for Environmental Determinants of Health (RCEDH), Health Institute, Kermanshah University of Medical Sciences Kermanshah Iran nazirfatahi@yahoo.com +988338263048 +989183364311

## Abstract

In this research, an environmental friendly, green and efficient sample preparation method using vortex-assisted microextraction based on a deep eutectic solvent (VAME-DES) followed by graphite furnace atomic absorption spectrometry (GFAAS) was developed for the preconcentration and determination of As(iii)/As(v) and total inorganic arsenic in soil and vegetables irrigated with treated municipal wastewater from Tehran and Kermanshah, Iran. In the proposed method, a novel DES, characterized by its low density, was prepared by mixing choline chloride and citric acid monohydrate at a molar ratio of 1 : 1. Under optimal conditions, the proposed method enabled the achievement of a good enrichment factor of 175. The calibration graph was linear in the range of 0.3–100 μg kg^−1^ and the limit of detection (LOD) was 0.10 μg kg^−1^. The repeatability and reproducibility of the method based on seven replicate measurements of 50 μg kg^−1^ As(iii) in analysed samples were 4.2% and 6.5, respectively. The relative recoveries from soil and vegetables that were spiked with different levels of As(iii) and As(v) were 94.2–104.3 and 91.0–107.0%, respectively. The main advantage of the proposed method is the use of a non-toxic and non-volatile DES instead of volatile organic solvents. The accuracy of the proposed procedure was also assessed by the speciation of arsenic in two standard reference materials (GBW10014 cabbage and GBW10015 spinach). The extraction methodology is simple, rapid, cheap and green, since only small amounts of non-toxic solvents are necessary.

## Abstract 2

An efficient method using vortex-assisted microextraction based on a deep eutectic solvent followed by graphite furnace atomic absorption spectrometry was developed for the determination of arsenic species in soil and vegetables irrigated with treated wastewater.

## Graphical abstract

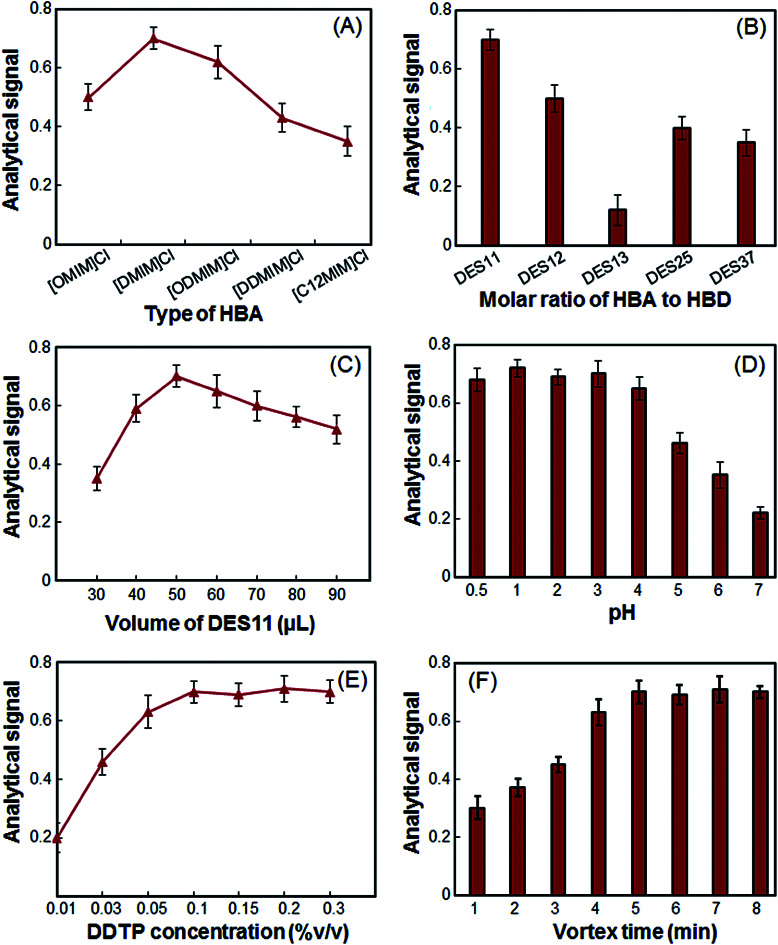

## 1 Introduction

In countries located in arid and semi-arid regions of the world, one of the most important problems facing agriculture as a major consumer of water resources is finding new and reliable water sources for irrigation. The use of wastewater for irrigation in agriculture in areas facing stress and water scarcity reduces the pressure on existing water resources and allows the allocation of high-quality water resources for other uses and the development of safety and health infrastructure.^1^ Therefore, considering recent droughts and the scarcity and reduction of high-quality water resources in Iran, irrigation with treated sewage or so-called wastewater has become a common strategy. Although the use of treated sewage for irrigation is a valuable way to increase the existing water resources, the quality and conditions of this water pose challenges to agriculture.^2^ Long-term use of sewage in land irrigation often results in increased levels of heavy, toxic and unnecessary metals in the soil.^3^ The accumulation of these metals in the soil not only reduces soil fertility and crop quality but also impairs the ecological role of soil and its impact on other environmental components.^4^ When the soil's capacity to retain metals decreases due to the increase of the surface of the soil, these metals are released as usable solutions for plant absorption.^5^ In fact, the main issue in the use of sewage in irrigation is the presence of toxic and hazardous metals in the sewage, their deposition in the soil and eventually their absorption by plants. In Iran, much of the water used in major cities such as Tehran and Kermanshah is converted into sewage, and due to the lack of water resources in the country, it is used for irrigation of agricultural lands after treatment.

The presence of arsenic in food and water has been considered a major risk factor by researchers. It exists in organic and inorganic forms both naturally and arising from human activities in the environment.^6^ Arsenic exposure can have adverse effects on human health and other organisms and can cause various side effects including skin changes, respiratory problems, cardiovascular disease, digestive system problems, genotoxicity and mutagenic and carcinogenic effects.^7^ Arsenic in nature has four oxidation modes including As(0), As(iii), As(v) and As(−iii). The degree of mobility and toxicity of arsenic depends on these oxidation modes.^8^ As(v) and As(iii) are classified as group I carcinogens by IARC.^9^ Speciation of iAs is often as important as total quantification because of its varying degrees of toxicity.^10^ High concentrations of arsenic in agricultural soils irrigated with treated sewage not only reduce the quality and efficiency of agricultural products but also endanger human health. Vegetables as an important component of the human diet can absorb arsenic from contaminated agricultural soil and accumulate it in its edible parts.^11^ As one of the important factors affecting human health is the quality of agricultural products, the development of sensitive, rapid and low cost techniques for measuring and tracking arsenic in these products is very important.

Nowadays, many modern instrumental techniques including electrothermal atomic absorption spectrometry (ETAAS),^12–14^ inductively coupled plasma optical emission spectrometry (ICP-OES),^15,16^ inductively coupled plasma mass spectrometry (ICP-MS),^17,18^ atomic fluorescence spectrometry (AFS)^19^ and electrochemical analysis^20^ have been used for the determination of low levels of arsenic. ETAAS is still being used because it combines a short analysis time, low detection limit, low sample volume requirements, simplicity and lower cost. However in this technique, complexity of matrices and low concentrations of analytes are the main problems. Accordingly, a suitable preconcentration step prior to instrument detection is necessary. For the extraction of arsenic in different samples, a variety of methods have been used so far, including liquid–liquid extraction (LLE),^21^ solid phase extraction (SPE),^22,23^ cloud point extraction (CPE),^24^ solid-phase microextraction,^25,26^ dispersive liquid–liquid microextraction (DLLME)^27–29^ and dispersive liquid–liquid microextraction based on solidification of a floating organic drop (DLLME-SFO).^30–32^ Advantages and disadvantages of these techniques have already been discussed.^33,34^

In recent years, affordable and green extractants, called deep eutectic solvents (DESs), are being used as an alternative to organic toxic solvents and ionic liquids for the process of separation and preparation of different samples.^35–38^ A DES is composed mainly of two or more components, and a eutectic mixture with a melting point lower than of all its constituents forms itself through hydrogen bonding. DESs not only have the advantages of high thermal stability, low volatility, low vapor pressure and a high ability to extract different analytes, but also are inexpensive and easily prepared non-toxic and safe compounds.

In the present study, a new DES was applied to the extraction and preconcentration of As(iii) and As(v) species in soil and vegetables irrigated with treated municipal wastewater from Tehran and Kermanshah, Iran prior to their analysis by modified tube graphite furnace atomic absorption spectrometry (GFAAS). In this method, a mixture of choline chloride (ChCl) and citric acid monohydrate is selected as a deep eutectic solvent in a molar ratio of 1 : 1. The total inorganic arsenic was measured after reduction of As(v) to As(iii) with Na_2_S_2_O_3_ and KI, and the concentration of As(v) was calculated by subtracting the As(iii) concentration from the total arsenic concentration.

## 2 Experimental

### 2.1 Reagents and solutions

Stock standard solutions of As(iii) and As(v) were prepared by dissolving appropriate amounts of As_2_O_3_ and Na_2_HAsO_4_·7H_2_O, respectively (Merck, Darmstadt, Germany). Working standard solutions were obtained daily by diluting the stock solution with ultrapure water. A chemical modifier solution for GFAAS was prepared by using a mixture of Pd(NO_3_)_2_ (1000 mg L^−1^) and Mg(NO_3_)_2_ (300 mg L^−1^) solutions, both from Merck (Darmstadt, Germany). Choline chloride and citric acid monohydrate both with a purity higher than 99% were purchased from Sigma-Aldrich (St. Louis, Missouri, USA). The chelating agent, diethyldithiophosphoric acid (DDTP) with a density of 1.17 kg L^−1^ was purchased from Merck. Na_2_S_2_O_3_ and KI (both Merck) were added for the reduction of As(v) to the trivalent state in sample solutions in order to determine total As.

### 2.2 Instrumentation

Measurements were carried out on a Model nov AA 400 atomic absorption spectrometer (Analytik Jena AG, Jena, Germany), equipped with a graphite furnace, auto-sampler MPE-60 and deuterium lamp for background correction. Pyrolytic graphite coated graphite tubes with an integrated PIN platform (Analytik Jena part no. 407-A81.026) were used. Argon 99.999% (Roham Gas Co., Arak, Iran) was used as a protecting and purging gas. Integrated absorbance (peak area) was used exclusively for signal evaluation and quantification. The optimum operating parameters for GFAAS are given in Table 1. A microwave closed system Multiwave 3000 (Anton Paar, Germany) was used for digestion of samples. The pH values were measured with a Metrohm pHmeter (Model: 692, Herisau, Switzerland) supplied with a glass-combined electrode. The Hettich Zentrifugen (EBA20, Tuttlingen, Germany) was used for centrifugation.

Instrumental parameters and temperature programming for the determination of arsenic

**Table:** 

Spectrometer parameters	
Wavelength (nm)	193.7
Spectral bandwidth (nm)	0.8
Lamp current (mA)	5.0

a Auto-zero.

**Table:** 

Step	Temperature (°C)	Ramp time (s)	Hold time (s)	Argon flow rate (L min^−1^)
Inject modifier	80	5	25	2
Inject sample				
Drying I	110	4	20	2
Drying II	240	3	12	2
Pyrolysis	750	15	10	2
AZ^a^	700	0	5	0
Atomization	2000	0	3	0
Cleaning	2400	0	3	2

### 2.3 Sampling

Four soil samples from different fields (two of Kermanshah and two of Tehran, Iran) were randomly collected at a depth of 5 to 20 cm with a stainless steel auger. The soil collected from each field was dried and sieved with a 0.2 mm sieve. The soil samples were then stored in brown glass bottles and placed in a desiccator in order to avoid exposure to light and moisture until required for analysis. Four vegetable cultivars including radish (*Raphanus sativus*), spinach (*Spinacia oleracea*), coriander (*Coriandrum sativum*) and carrot (*Daucus carota*), were collected from the same locations simultaneously with the soils. The collected vegetable samples were washed thoroughly with tap water and then rinsed two times with distilled water to remove dust. The edible parts of the vegetable samples were weighed and then oven-dried in a hot air oven at 75–80 °C for 24 h to remove moisture. The dried samples were ground with an agate mortar and passed through a 0.2 mm sieve to obtain a uniform size. All homogenized sample powders were stored in brown glass bottles and preserved in a desiccator before analysis.

### 2.4 Sample preparation

Half a gram of each soil sample was placed in a 20 mL digestion tube and 8.0 mL of concentrated HNO_3_ was added. The samples were digested with a MARS X-Press (CEM Corporation, NC, USA) microwave oven after the application of a preselected program: first stage: power = 800 W; ramp time (min) = 1; hold time (min) = 3 min; temp (°C) = 75 and second stage: power = 800 W; ramp time (min) = 1; hold time (min) = 4 min; temp (°C) = 85. After cooling, 12 mL of HClO_4_ (70%) was added and the mixture was boiled gently until the appearance of dense white fumes. Samples were allowed to cool down to room temperature and the contents were transferred to 20 mL centrifuge tubes, which were then centrifuged at 5000 rpm for 5 min at room temperature. The supernatant was transferred to a glass test tube and its pH was adjusted to 3 with sodium hydroxide (2 mol L^−1^). An aliquot of 10 mL of the resulting solution was subjected to the VAME-DES procedure.

For the digestion of vegetables samples, 1.0 g of the sample was accurately weighed and digested with 2.0 mL of HClO_4_ and 8.0 mL of HNO_3_. The samples were allowed to cool and the contents were filtered off using Whatman no. 42 filter paper. The filtrate was made up to 20 mL with distilled water. Finally, 10 mL of this sample solution was subjected to the VAME-DES procedure.

### 2.5 Preparation of hydrophobic DESs

To synthesize DES, five imidazolium chlorides as HBA and citric acid monohydrate as HBD were mixed with a 1 : 1, 1 : 2, 1 : 3, 2 : 5 and 3 : 7 ratio and stirred very well. The resulting mixture was shaken at 600 rpm speed at 70 °C until it became clear and complete colorless. The DES were then stored in a desiccator to prevent moisture absorption. After that, DES was cooled until it was room temperature and used as the extraction phase in the VAME-DES method to extract arsenic ions.

### 2.6 VAME-DES procedure

For the presented procedure, an aliquot of 10.0 mL of ultra-pure water or sample solution spiked or not with As(iii) was placed in a 20 mL test tube and 50.0 μL of DES as the extraction solvent containing 10.0 μL DDTP (chelating agent) was rapidly injected into the sample solution with a 100 μL syringe (Gastight, Hamilton, Reno, NV, USA). The mixture was then shaken using a vortex agitator for 5 minutes to ensure full contact of the DES and arsenic ions from the sample solution, and finally centrifuged at 5000 rpm for 4 min. After centrifugation, the fine droplets of DES floated at the top of the tube. The tube was then transferred into a freezer and the DES was solidified after 5 min. Then the obtained solidified DES was transferred into a conical vial where it was melted immediately. Finally, for quantitation of As(iii), 30.0 μL of the extract using an auto-sampler was injected into the GFAAS and was subjected to the temperature program, shown in Table 1.

## 3 Results and discussion

### 3.1 Selection of the deep eutectic solvent

**Fig. 1:**
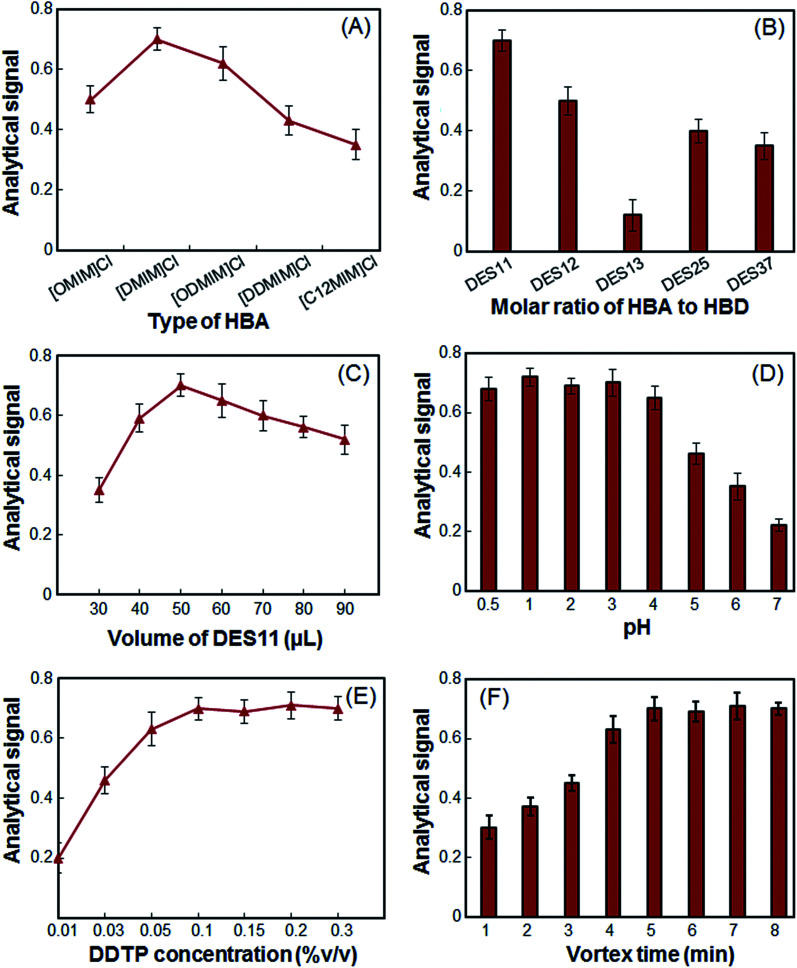
The effects of (A) different types of HBA, (B) the molar ratio of HBA to HBD, (C) the volume of DES11, (D) the sample solution pH, (E) the concentration of DDTP and (F) the vortex time on the absorbance of arsenic.

In the present study, five imidazolium chlorides were chosen as HBAs and these imidazolium chlorides were mixed with citric acid monohydrate in a mole ratio of 1 to 1. Other experimental conditions were kept constant. As shown in Fig. 1(A), [DMIM]Cl as HBA, shows a higher analytical signal for extraction of As(iii). The analytical signal of other DESs for the extraction of As(iii) is not more than 0.5, because the dispersion of these DESs is not very strong in the absence of a disperser solvent and they do not disperse well. As a result, [DMIM]Cl was chosen as HBA.

### 3.2 Selection of HBA to HBD molar ratio

In the present work, the extractant was selected by mixing citric acid monohydrate and choline chloride ([DMIM]Cl) with different ratios of 1 : 1 (DES11), 1 : 2 (DES12), 1 : 3 (DES13), 2 : 5 (DES25) and 3 : 7 (DES37). The results are shown in Fig. 1(B). According to Fig. 1(B), the HBA and HBD do not form the DES well in a molar ratio of 1 : 3 because the obtained mixture is gelatinous and not well dispersed in the aqueous solution. Other molar ratios have a positive effect on the extraction of the As(iii). The combination of HBA and HBD at a 1 : 1 molar ratio has a lower standard deviation and better efficiency. As a result, the ratio of 1 : 1 was chosen as the optimal molar ratio.

### 3.3 Selection of the extraction solvent volume

In extraction methods, extractant volume could play two different roles. On the one hand, by increasing extractant volume, recovery of extraction could be increased as a result of increasing available solvent droplets. On the other hand, by further increasing the volume, extraction efficiency could be decreased as a result of the dilution effect. Accordingly, to select the optimum DES11 volume, several experiments were performed using different volumes of DES11, *i.e.* 30, 40, 50, 60, 70, 80 and 90 μL. As can be seen from Fig. 1(C), the analytical signal of the arsenic ions increased gradually up to 50 μL and then decreased as a result of the dilution effect. Therefore, 50 μL of the DES was chosen for further experiments.

### 3.4 Selection of sample solution pH

The sample solution pH is an important factor in microextraction of As(iii) using DDTP because it is directly related to the formation of As–DDTP species.^6^ In this study, DDTP was totally transformed to the DDTP ammonium salt with ammonia and the effect of pH on the formation of the metal ion complex was investigated in the range of 0.5–7, using HCl and CH_3_COONa solutions. As it is shown in Fig. 1(D), the analytical signal is nearly constant and shows a maximum in the pH range of 0.5–4, followed by a reduction at higher pH values. Since the nature of DDTP solution is acidic (pH 2.1 in 10.0 mL aqueous solutions), the use of secondary acidic solution for the pH adjustment, which are sources of contamination, is not necessary.

### 3.5 Selection of DDTP concentration

The DDTP was the best chelating agent for As(iii) extraction. Enough DDTP was needed to ensure that a lot of As(iii) was extracted. Hence, the DDTP concentration was investigated over the range of 0.01–0.50% (v/v). Fig. 1(E) illustrated that when the concentration of DDTP was up to 0.09% (v/v), most As(iii) ions were extracted, and with the further increase of the DDTP concentration, the absorbance had no significant increase. Therefore, the concentration of 0.10% (v/v) was chosen as the optimum concentration of DDTP for determination of As(iii) to prevent any interference.

### 3.6 Selection of vortex time

The effective vortex of the sample solution could lead to acceleration of the analyte transfer from the sample solution to the extraction phase. Initial experiments showed that in the absence of a vortex, dispersion of the DES in the sample solution is not good. However, in the presence of a vortex, the DES is completely dispersed in the sample solution. Accordingly, the vortex time was studied in the range of 1–8 min. The results shown in Fig. 1(F) revealed that the analytical signal of the As(iii) increased with increasing vortex time from 1 to 5 min and, with increasing vortex time, the analytical signal is constant. Thus, a 5 min vortex time was used in subsequent experiments.

### 3.7 Interference studies

The most common matrix constituents of real samples such as alkali and alkaline earths do not react with DDTP because of their selectivity.^34^ The potential interference of some ions on the preconcentration and determination of As(iii) was tested. The recovery of 5.0 μg L^−1^ of As(iii) solution in the presence of various amounts of interfering ions were calculated according to the presented procedure. The criterion for interference of each species was set at ±5.0% in the analytical signal obtained for a solution containing As(iii), without any interference. Table 2 shows the tolerance limits of the interfering ions.

**Table:** The effects of potentially interfering ions on the recovery of 5.0 μg L^−1^ As(iii)

Interferent	Interferent/As(iii) ratio	Recovery (%)
Na^+^	5000	96.5
K^+^	5000	95.0
Li^+^	5000	101.5
Ca^2+^	4000	97.0
Ba^2+^	2000	101.4
Mg^2+^	2000	94.6
Al^3+^	2000	103.4
Co(ii)	1000	99.5
Se(iv)	100	98.2
Sb(iii)	100	94.5
Fe(ii)	500	103.3
Fe(iii)	300	93.0
Ni(ii)	200	102.5
Zn(ii)	100	92.8
Pb(ii)	100	97.2
Cd(ii)	100	93.4
Cu(ii)	100	97.1
Cl^−^	5000	93.5
SO_4_^2−^	5000	101.5
NO_3_^−^	5000	100.8

### 3.8 Method performance

**Fig. 2:**
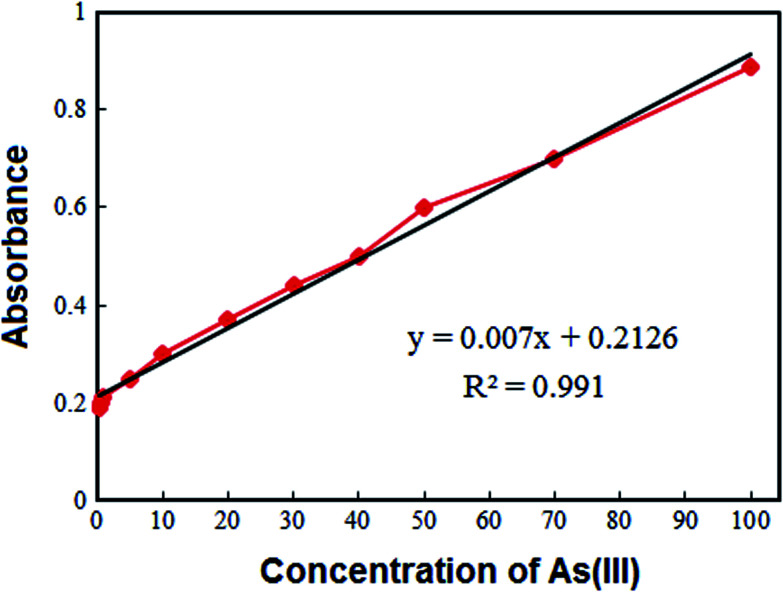
The calibration curve of As(iii) obtained under the optimized conditions.

The applicability of the VAME-DES method was examined for extraction and preconcentration of As(iii) from soil and vegetables. To evaluate the performance of the proposed technique, repeatability (intra-day), reproducibility (inter-day), linear dynamic ranges, limit of detections, enrichment factors and enhancement factors were investigated by utilizing standard solution of As(iii) in ultra-pure water and the obtained results are summarized in Fig. 2 and Table 3. The repeatability (intra-day) and reproducibility (inter-day) of the method were measured by executing seven replicate determinations of As(iii) at a 50 μg kg^−1^ concentration, that were 4.2% and 6.5%, respectively. The linear dynamic range was 0.30–100 μg kg^−1^ with a correlation coefficient (*r*^2^) better than 0.990. The detection limit, defined as *C*_L_ = 3*S*_b_/*m* (where *C*_L_, *S*_b_ and *m* are the detection limit, standard deviation of the blank and slope of the calibration curve, respectively), was 0.10 μg kg^−1^. The enrichment factor and enhancement factor were 175 and 128, respectively.

**Table:** The analytical characteristics of VAME-DES-GFAAS for the determination of As(iii) in soil and vegetables

Parameter	Analytical feature
Linear range (μg kg^−1^)	0.30–100
*r* ^2^	0.991
Limit of detection (μg L^−1^) (3*σ*, *n* = 7)	0.10
RSD%^a^ (intra-day, *n* = 7)	2.8
RSD% (inter-day, *n* = 7)	5.5
Enrichment factor	175

a As(iii) concentration was 50 μg kg^−1^.

### 3.9 Real sample analysis

The efficiency of the proposed VAME-DES method has been successfully applied to the extraction and pre-concentration of inorganic arsenic and total inorganic arsenic species. Soil and vegetable samples were selected from two farms in Tehran and two farms in Kermanshah and analyzed 24 hours after sampling. The analysis of each sample was repeated three times and the results are summarized in Table 4. As(iii) is directly analyzed by the GFAAS, but to measure As(v), first by reducing As(v) to As(iii) using sodium thiosulfate and potassium iodide, the total inorganic arsenic concentration is obtained, and then by subtracting the As(iii) concentration from total inorganic arsenic, As(v) is obtained. Concentrations of As(iii) and As(v) in soil samples ranged from 61.2–162.3 and 85.0–181.2 μg kg^−1^, respectively. Similarly, concentrations of As(iii) and As(v) in vegetables ranged from 32.3–155.0 and 19.5–123.7 μg kg^−1^, respectively (Table 4).

**Table:** Determination of As(iii), As(v) and total inorganic arsenic in soil and vegetables

Farmland no.	Sample	As(iii) concentration (μg kg^−1^) ± SD (*n* = 3)	As(v) concentration (μg kg^−1^) ± SD (*n* = 3)	t-iAs concentration (μg kg^−1^) ± SD (*n* = 3)
Kermanshah-1	Soil	88.5 ± 6.2	98.3 ± 6.8	186.8 ± 13.0
	Radish	51.2 ± 3.1	64.5 ± 4.6	115.7 ± 7.7
	Spinach	32.3 ± 2.6	19.5 ± 1.3	51.8 ± 3.9
	Coriander	97.3 ± 7.4	108.2 ± 7.3	205.5 ± 14.7
	Carrot	113.5 ± 8.5	96.7 ± 8.5	210.2 ± 17.0
Kermanshah-2	Soil	61.2 ± 4.2	85.0 ± 6.3	146.2 ± 10.5
	Radish	37.6 ± 2.2	53.5 ± 4.2	91.1 ± 6.4
	Spinach	58.2 ± 3.8	43.8 ± 2.8	102.0 ± 6.6
	Coriander	55.9 ± 4.4	72.0 ± 5.4	127.9 ± 9.8
	Carrot	86.2 ± 6.7	93.5 ± 7.6	179.7 ± 14.3
Tehran-1	Soil	162.3 ± 8.6	181.2 ± 13.6	343.5 ± 22.2
	Radish	82.5 ± 4.3	77.6 ± 4.8	160.1 ± 9.1
	Spinach	56.2 ± 3.5	49.7 ± 3.2	105.9 ± 6.7
	Coriander	108.7 ± 7.2	112.5 ± 7.4	221.2 ± 14.6
	Carrot	155.0 ± 11.3	123.7 ± 10.6	278.7 ± 21.9
Tehran-2	Soil	122.4 ± 10.5	151.5 ± 11.3	273.9 ± 21.8
	Radish	44.8 ± 2.7	37.3 ± 2.6	82.1 ± 5.3
	Spinach	82.3 ± 5.4	95.2 ± 6.2	177.5 ± 11.6
	Coriander	48.2 ± 3.2	55.6 ± 4.0	103.8 ± 7.2
	Carrot	90.5 ± 6.5	118.3 ± 8.7	208.8 ± 15.2

To evaluate the validity of the proposed method, one soil sample and two vegetable samples harvested from Kermanshah farms and one soil sample and two vegetable samples harvested from Tehran farms were spiked at different concentration levels with As(iii) and As(v) and relative recoveries were calculated. The results in Table 5 show the relative recoveries of As(iii) and As(v) in the different samples were in the range of 91.5–106.3 and 91.0–107.0%, respectively. The accuracy of the proposed procedure was also assessed by determining the concentration of the total inorganic arsenic in two standard reference materials (GBW10014 cabbage and GBW10015 spinach). As can be seen from Table 5, the obtained values are in satisfactory agreement with the certified values. These results showed that the matrices of the analyzed real soil and vegetables have little effect on ME-DES followed by GFAAS for determination of inorganic arsenic species.

**Table:** The relative recoveries and standard deviations of As(iii) and As(v) from spiked soil and vegetables

Farmland no.	Sample	Analyte	Added (μg kg^−1^)	Found (μg kg^−1^) ± SD (*n* = 3)	Relative recovery (%)
Kermanshah-1	Soil	As(iii)	0	88.5 ± 6.2	—
			50	135.6 ± 9.7	94.2
		As(v)	0	98.3 ± 6.8	—
			50	146.2 ± 10.3	95.8
	Radish	As(iii)	0	51.2 ± 3.1	—
			30	83.1 ± 6.4	106.3
		As(v)	0	64.5 ± 4.6	—
			30	95.7 ± 5.8	104.0
	Spinach	As(iii)	0	32.3 ± 2.6	—
			20	50.6 ± 4.5	91.5
		As(v)	0	19.5 ± 1.3	—
			20	38.3 ± 2.1	94.0
Tehran-2	Soil	As(iii)	0	122.4 ± 10.5	—
			60	185.0 ± 11.6	104.3
		As(v)	0	151.5 ± 11.3	—
			60	210.2 ± 14.8	97.8
	Coriander	As(iii)	0	48.2 ± 3.2	—
			10	57.8 ± 3.5	96.0
		As(v)	0	55.6 ± 4.0	—
			10	64.7 ± 3.8	91.0
	Carrot	As(iii)	0	90.5 ± 6.5	—
			40	132.3 ± 9.6	104.5
		As(v)	0	118.3 ± 8.7	—
			40	161.1 ± 11.3	107.0
SRM, GBW10014	Cabbage	t-iAs	0.062 ± 0.014^a^	0.065 ± 0.09	104.8
SRM, GBW10015	Spinach	t-iAs	0.23 ± 0.03^a^	0.21 ± 0.02	91.3

a Certified values (μg g^−1^).

### 3.10 Comparison of VAME-DES with previously reported methods

The presented method was compared with some of the methods that were recently published in the literature for preconcentration and determination of the arsenic in different samples. The results are summarized in Table 6. Based on the data, the LDR and LOD of the proposed method are similar or better than other methods. Also, the proposed method as with other microextraction methods is associated with advantages such as rapidity, a high enrichment factor, simplicity, high efficiency, and high recovery and low consumption of solvents and reagents. The extraction time in this method is shorter than in other methods, except for the conventional DLLME method. However, unlike the DLLME method, a disperser solvent is not required in this method, and the amount of organic solvent used is very low.

**Table:** A comparison of VAME-DES with other extraction methods for the determination of arsenic in different samples

Method	LOD^a^ (μg kg^−1^)	LR^b^ (μg kg^−1^)	RSD%^c^	Extractant volume (μL)	Sample amount (g)	Sample	Reference
ETA-MILs-ME-GFAAS^d^	7	0.02–10	2.9	65	0.5	Vegetables	8
EEM-ICP-MS^e^	8	—	<9	10 000	0.5	Staple diets	5
CL-DES-MNF-AALLME-ETAAS^f^	0.036	0.005–0.1	3.1	40	2	Food samples	10
DSLLME-ETAAS^g^	0.02	0.08–2	5.3	1030	5 mL	Environmental water	30
CCLLME-ETAAS^h^	0.03	0.1–50	2.3	30	2–4 mL	Biological fluids	34
MADLLME-ETAAS^i^	0.2	0.5–200	5.3	650	0.25	Rice	6
ME-DES-GFAAS	0.1	0.3–100	4.2	50	0.5–1	Soil and vegetables	This work

a LOD: limit of detection.

b LR: linear range.

c RSD: relative standard deviation.

d Tablet-assisted magnetic ionic liquid-based microextraction and graphite furnace atomic absorption spectrometry.

e Enzymatic extraction methods.

f Centrifugeless deep eutectic solvent based magnetic nanofluid-linked air-agitated liquid–liquid microextraction and electrothermal atomic absorption spectroscopy.

g Dispersive-solidification liquid–liquid microextraction and electrothermal atomic absorption spectroscopy.

h Countercurrent liquid–liquid microextraction and electrothermal atomic absorption spectroscopy.

i Microwave assisted dispersive liquid–liquid micro-extraction and electrothermal atomic absorption spectrometry.

## 4 Conclusions

In this work, a vortex-assisted microextraction method with a new deep eutectic solvent was applied to the extraction and preconcentration of inorganic arsenic in soil and vegetables irrigated with treated municipal wastewater. The new DES consists of [DMIM]Cl and citric acid monohydrate parts at a molar ratio of 1 : 1. DES11 was rapidly injected into the aqueous solution and a cloudy system was formed, which resulted in the rapid extraction of the complexed ions due to contact between the extractant and the aqueous solution. In addition, the use of the vortex as a dispersing agent eliminates the use of toxic solvents, such as acetone, methanol or acetonitrile, in conventional DLLME and the organic solvent consumption is very low. The modifications introduced in the proposed method allow it to be classified as environmentally friendly. In the end, the VAME-DES-GFAAS method was successfully applied to the speciation of arsenic in soil and vegetables with good precision, quantitative recovery and a high enrichment factor.

## Conflicts of interest

There are no conflicts to declare.

## Supplementary Material
